# Does Direct and Indirect Exposure to Ionising Radiation Influence the Metastatic Potential of Breast Cancer Cells

**DOI:** 10.3390/cancers12010236

**Published:** 2020-01-17

**Authors:** Munira A. Kadhim, Ammar Mayah, Susan A. Brooks

**Affiliations:** Department of Biological and Medical Sciences, Faculty of Health and Life Sciences, Oxford Brookes University, Oxford OX3 0BP, UK; amayah@brookes.ac.uk (A.M.); sbrooks@brookes.ac.uk (S.A.B.)

**Keywords:** ionising radiation, glycosylation, epithelial mesenchymal transition, EMT, exosomes, invasion, metastasis

## Abstract

Ionising radiation (IR) is commonly used for cancer therapy; however, its potential influence on the metastatic ability of surviving cancer cells exposed directly or indirectly to IR remains controversial. Metastasis is a multistep process by which the cancer cells dissociate from the initial site, invade, travel through the blood stream or lymphatic system, and colonise distant sites. This complex process has been reported to require cancer cells to undergo epithelial-mesenchymal transition (EMT) by which the cancer cells convert from an adhesive, epithelial to motile, mesenchymal form and is also associated with changes in glycosylation of cell surface proteins, which may be functionally involved in metastasis. In this paper, we give an overview of metastatic mechanisms and of the fundamentals of cancer-associated glycosylation changes. While not attempting a comprehensive review of this wide and fast moving field, we highlight some of the accumulating evidence from in vitro and in vivo models for increased metastatic potential in cancer cells that survive IR, focusing on angiogenesis, cancer cell motility, invasion, and EMT and glycosylation. We also explore the indirect effects in cells exposed to exosomes released from irradiated cells. The results of such studies need to be interpreted with caution and there remains limited evidence that radiotherapy enhances the metastatic capacity of cancers in a clinical setting and undoubtedly has a very positive clinical benefit. However, there is potential that this therapeutic benefit may ultimately be enhanced through a better understanding of the direct and indirect effects of IR on cancer cell behaviour.

## 1. Introduction

Breast cancer is the most common cause of cancer-related death in women worldwide. The major risk factors are related to reproductive biology, for example, early age at menarche and late menopause, older age at first full term pregnancy or nulliparity, and use of hormone-based medication. However, it has well been established that ionising irradiation can also be implicated in breast cancer induction. Exposure to ionising radiation (IR) has greater effects on women in childhood and adolescence than adulthood [1]. IR-induced breast cancer is frequently higher in women who were exposed to IR when they were younger than 20 years compared to women exposed at older ages. Women exposed to IR when older than 50 years show no significant increase in breast cancer risk following irradiation [2]. The development of breast tissues is different from other organ tissues because in the breast, proliferation and growth can rapidly happen when it is prepared during a first full term of pregnancy [3]. Mammary carcinogenic risk and susceptibility often increase during the cell proliferation period [4,5], during which DNA synthesis and replication also increase. Consequently, this can lead to a higher chance of DNA damage to the offspring cells [6]. Furthermore, DNA double strand break repair mechanisms are often mediated by BRCA1 and BRCA2 and mutation of these genes has been shown to significantly increase breast cell radiosensitivity in some studies [7,8,9,10,11,12,13,14,15], although this is not established. One of the keystone breast cancer therapeutic techniques is radiotherapy (RT), during which there is an aim to diminish the damaging effects to neighbouring normal tissues over cancer cells [16,17]. RT outcome is clinically based on radiation type, doses, fractions, tumour replication time, hypoxia, and radiosensitivity of the tumour [18].

## 2. The Role of Signalling Molecules and Radiation Response

Communication between irradiated and non-irradiated neighbouring cells (bystander effects) or out-of-field cells (abscopal effects) can cause cellular damage and underlies non-targeted effects of IR (NTE) [19]. Cytokines and chemokines, such as interleukin (IL)-1, 2, 6, 8, 10 and TGF-β, play a crucial role in cell–cell communication as they are normally secreted in the microenvironment. Interestingly, a high level of IL-1β is observed in ductal breast carcinoma, while normal tissue does not show any overexpression of IL-1β [20]. Evidence suggests that a small amount of IL-1 can potentially cause other cytokines to be secreted from other cells [21]. Moreover, proliferation, invasion, angiogenesis, and cancer cell apoptotic inhibition are highly associated with IL-1 overexpression [22,23]. Breast cancer aggressiveness can be mediated by IL-1α and IL-8 by increasing metastasis and cachexia [24,25]. It has also been well established that oestrogen activity and oestrogen receptors can be controlled by IL-1 family members. Hence, oestrogen receptor negative breast cancer cells show a high level of IL-1 [26]. In addition, breast cancer tissue secreted-IL-8 can promote endothelium proliferation, cancer cell survival, angiogenesis, and matrix metalloproteinase (MMP) production [27,28,29]. The role of the IL-1 family is based on the association of family members with prognostic indicators. Human breast cancer tissue can express IL-1 α and β (IL-1 pro-inflammatory agonists) and IL-1receptor antagonists. Both IL-1 α and β are able to regulate tumour cell proliferation and control tumourigenic factor production, such as the production of angiogenic and growth factors. The levels of IL-1 α and β correlate with tissue levels of IL-8, which is an angiogenic factor [20]. Moreover, breast fibroblast cells secrete IL-6, which can increase proliferation and invasiveness of oestrogen receptor positive cells, such as breast cancer MCF7 cells [30,31]. Epithelial-mesenchymal transition (EMT), discussed later, can be mediated by the overexpression of IL-6 [32]. Breast cancer patients showed higher levels of IL-6 in their serum than healthy people [33]. Conversely, IL-24, which is a cytokine of the IL-10 family, has the ability to suppress vascularisation of tumours [34]. Moreover, an increase in IL-10, which is an anti-inflammatory cytokine, can reduce the levels of pro-inflammatory cytokines, such as TNF-α, IL-6, and IL-1β [35,36].

Radiotherapy primarily increases cytokine production in breast cancer patients, including IL-2, 6, 8, and 10. There is a high induction of IL-2 in the breast cancer patients’ sera after one month following RT; however, this level returns to baseline after six months post irradiation [37]. IL-6 level is also higher in radiotherapy-treated breast cancer patient’s plasma compared to unexposed patients [38]. Schmidt and co-authors have also shown that IL-6 induction is higher during the early period of RT (three weeks post irradiation) compared to levels in patients at six weeks into treatment [39]. Similarly, Westbury et al. documented that IL-6 level serum is an early time-dependent biomarker and also demonstrated no significant difference in breast cancer patients’ IL-6 serum levels IL-6 two or five years following RT [40].

TGF-β1 also has a direct pathophysiological effect on breast cancer; it can play a crucial role in EMT increasing tumour motility and invasion [41]. It can also enhance angiogenesis and progression [42]. However, TGF-β1 has also been shown to be a growth suppressor, inhibiting the progression of epithelial cell cycle and causing apoptosis. Consequently, the latter two effects play an important role in tumour suppression, in particular, during carcinoma initiation, promotion, and progression stages [43]. RT can increase TGF-β levels in the cancer patients’ sera. Boothe et al. reported that TGF-β1 serum levels of breast cancer patients can be used as an early biomarker for radiation-induced fibrosis post RT [44]. Evidence was also found for a high level of TGF-β1 in the breast cancer patients’ wound fluids 24 h following surgery [45].

## 3. Metastasis

**Figure 1:**
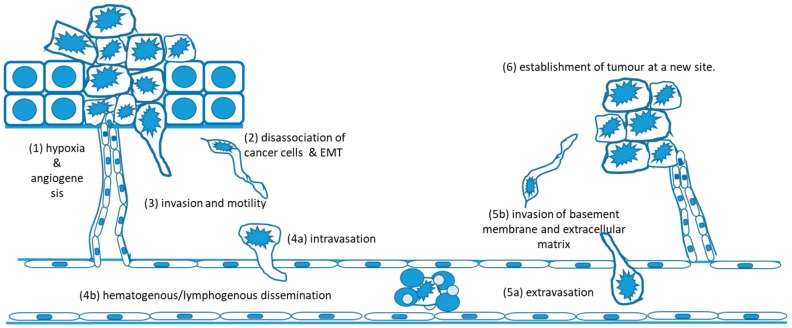
The metastatic cascade: (1) hypoxia in the growing tumour stimulates new blood vessel formation (angiogenesis) to supply the tumour with oxygen and nutrients and remove waste products. (2) Hypoxia is also one of the signals that induces epithelial-mesenchymal transition (EMT) whereby rounded, adherent epithelial cancer cells break cell–cell and cell–basement membrane contacts and change to a motile, mesenchymal phenotype. (3) Cancer cells produce matrix degrading enzymes and migrate through the stroma towards blood vessels or lymphatics (4a) cancer cells enter blood vessels or lymphatics. (4b) They are transported through blood/lymph in blood vessels often forming protective aggregates with leukocytes and platelets (5a) at a distant site, cancer cells may become mechanically trapped in small vessels and/or may actively adhere to endothelium, (5b) they then extravasate and invade through basement membrane surrounding vessels and through local stroma using similar molecular mechanisms to those employed in step 3. (6) For successful establishment of a tumour at the new site, cancer cells must once again stimulate angiogenesis, as in step 1, and adapt to and flourish in a new environment.

Despite the effectiveness of established therapies such as radiotherapy, metastasis remains the most important clinical issue in cancers, including breast cancer. The majority—around 90%—of deaths from cancer result not from the original primary tumour, but from metastatic disease [46]. Metastasis is the process by which cancer cells dissociate from the primary tumour, become motile, and travel through the lymphatics or bloodstream to distant anatomical sites where they establish new tumours. This disseminated disease is usually widespread and multifocal, is frequently not detected until the tumours are relatively large and well established, is often resistant to treatments, including radiotherapy, and is difficult, if not impossible, to fully eradicate. It therefore represents a major clinical challenge in cancer therapy. The process of metastasis is a complex one, involving a myriad of molecular players, and can, for convenience, be described in a number of steps [47,48], which are outlined briefly below and illustrated in Figure 1:

### 3.1. Hypoxia and Angiogenesis

Early in its genesis, a cancer receives oxygen and nutrients and disposes of its waste products through simple diffusion from the nearest blood vessel(s). This process is efficient as long as the tumour reaches a size no larger than around 2 mm in diameter. Once it begins to grow larger, parts of the tumour mass—particularly near the centre—become hypoxic and the hypoxia triggers a complex cascade of molecular events that result in the stimulation of angiogenesis—the growth of new blood vessels to supply the tumour [49,50,51]. In healthy tissue, the process of angiogenesis is very tightly controlled (e.g., during development and wound healing) and results in a highly organised neovasculature. In tumour angiogenesis, the process is disregulated and results instead in the formation of poorly formed, tortuous, dead-ended and leaky blood vessels, which themselves may help facilitate metastasis.

### 3.2. Disassociation of Cancer Cells from the Primary Tumour Mass and Epithelial-Mesenchymal Transition (EMT)

Hypoxia is also amongst the signals that lead to tumour cells undergoing dramatic changes in morphology and behaviour [52]. Within the primary tumour mass, cells of a cancer of epithelial origin, such as breast cancer, will themselves be epithelial in appearance. Typically, such cells have a rounded morphology, exhibit polarity, and are tightly adherent to neighbouring cells and/or to basement membrane. In order to metastasise, such cells need to loosen their contacts with their surroundings, change to a more mesenchymal or fibroblast-like morphology, and become motile. This process is referred to as “epithelial-mesenchymal transition” or EMT. At a molecular level, these dramatic changes are orchestrated largely by members of the SNAIL superfamily of transcription factors, principally Snail1/Snail2 and Slug. Their activation leads to the suppression of epithelial markers (including E-cadherin) and upregulation of mesenchymal markers (including N-cadherin, vimentin, fibronectin, and vitronectin) [53,54,55]. These changes result in reduced cell adhesion and increased motility. For example, downregulation of E-cadherin causes loss of cancer cell polarity and a reduction in cell–cell attachment [55]. Increases in the cytoskeletal element vimentin and the adhesion molecule N-cadherin both promote morphological change from epithelial to mesenchymal form, rendering cells more motile and invasive [56]. In addition, SNAIL-induced EMT renders cells more resistant to apoptosis [57].

### 3.3. Invasion of the Basement Membrane and Extracellular Matrix and Motility 

Cancer cells need to develop mechanisms other than EMT, including the production of proteolytic enzymes and transcription factors, in order to physically invade the basement membrane and extracellular matrix. The best characterised of the matrix degrading enzymes are the matrix metalloproteinases (MMPs), a family of endopeptidases which can degrade almost all components of the basement membrane and extracellular matrix [58]. Members of this family are frequently upregulated in cancer and promote tumour cell migration [59].

Cancer cells use a variety of mechanisms in order to migrate and invade and these are reminiscent of normal mechanisms employed in, for example, embryonic development, immune cell trafficking, or wound healing. There is evidence that in some cases, single cancer cells move in an amoeboid fashion and that in other instances, groups of cancer cells may act together to degrade surrounding extracellular matrix and migrate collectively [60,61].

### 3.4. Intravasation and Hematogenous/Lymphogenous Dissemination

In order to escape from their immediate surroundings and metastasise to distant sites, cancer cells must make their way into local lymphatics or blood vessels, a process termed intravasation. There is evidence that this can be achieved in two ways: an active mechanism whereby cancer cells utilise elements of the cytoskeleton (laminin, vimentin) to produce pseudopodia/lamellipodia and direct cells toward the endothelial cells of capillaries [62], or passively as a result of the pressures of growing cancer cells mechanically damaging vessels [63]. The flawed construction of the tumour-induced neovasculature, described previously, helps to facilitate this process.

Once they have made their way into vessels, the majority of cancer cells die as a result of mechanical trauma, immune attack, hypoxia, or anoikis [64]. However, some will survive and there is evidence that their chance of survival is improved when they form aggregates with other cancer cells, platelets, and leukocytes [65,66], perhaps because they are protected from immune attack or perhaps as a result of physical cushioning from the shear forces of blood flow.

### 3.5. Extravasation and Invasion of the Basement Membrane and Extracellular Matrix 

Controversy has existed for at least 130 years as to whether metastases formed by a primary tumour are as a result of the cancer cells in circulation specifically interacting and adhering to the endothelial lining of blood vessels in a distant site (the “seed and soil” hypothesis of Paget proposed in 1889) or whether they simply result from the mechanical trapping of cancer cells or emboli in the small blood vessels within organs [67]. There is ample evidence in the literature of mechanical entrapment of cancer cells within small vessels. However, there are also many studies that suggest that cancer cells also need to be able to specifically interact with the vessel walls in order to adhere and to extravasate, and some provide evidence of a specific metastatic niche. 

One theory is that cancer cells may utilise analogous mechanisms to those employed during leukocyte trafficking. Here, cytokines direct circulating leukocytes to sites of inflammation where they undergo, initially, weak selectin-mediated adhesion to the endothelial cells lining the vessels and subsequently firm adhesion mediated by integrins and members of the immunoglobulin superfamily [47]. Once adherent, leukocytes extravasate by two method—either by signalling to the endothelial cells to break cell-cell junctions and retract allowing passage through the gap created, or by a transcellular mechanism [68]. It is reported that cancer cells also extravasate using both of these routes [69] and they can also permanently physically damage the endothelium to create a break through which to escape [70]. Clearly, here the underlying mechanisms that support cancer cell motility, described earlier, also come in to play, facilitating this egress.

### 3.6. Establishment of Tumour at a New Site

Once cancer cells successfully extravasate at a distant site, it is believed that they revert to their original epithelial phenotype, termed mesenchymal-epithelial transition, MET [71]. Clearly, the new microenvironment will be very different from the one that the cancer cell left at the primary site and was adapted to. The challenge of the new environment means that most disseminated cancer cells either perish or lie dormant, sometimes for many years, until it or its progeny adapt or the environment changes [72,73,74]. The circumstances that cause apparently dormant micrometastases to begin to flourish—the underlying cause of diseases such as breast cancer recurring at distant sites years and sometimes decades after apparently successful initial treatment—is still not understood.

## 4. Potential Involvement of Glycosylation in Metastasis 

It is clear from the previous description of the process of metastasis that it is extremely complex and involves the interplay of a huge variety of molecular players. Unsurprisingly, it has been estimated, based on clinical observations and on animal models, that the metastatically successful cancer cell is actually very rare. Perhaps as few as 0.01% of cancer cells that successfully enter the blood circulation ultimately form a metastatic tumour [75].

**Figure 2:**
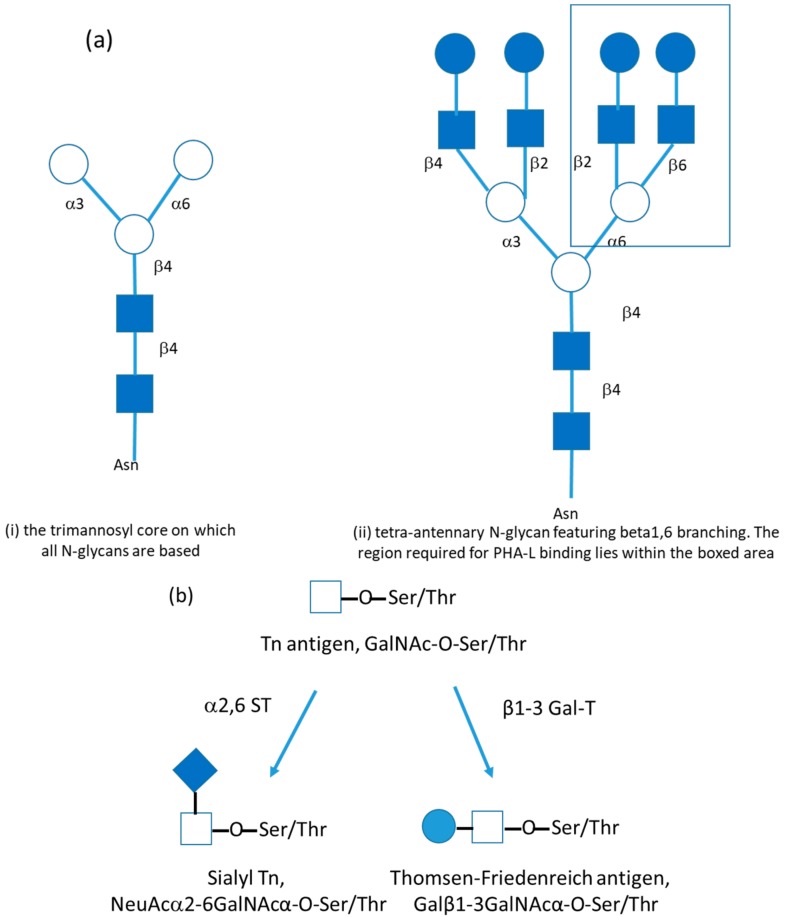
Examples of cancer-associated glycosylation changes (**a**), (i) N-linked glycans are based on a trimannosyl core linked to an asparagine (Asn) residue on the polypeptide. This can be extended by sequential addition of monosaccharides to form a huge variety of complex bi-, tri- tetra-, and penta-antennary structures. (ii) In cancer, N-glycans tend to be larger and more branched than in normal cells. One commonly reported finding is an increase in beta 1,6 branched glycans that can be detected by the binding of a lectin, PHA-L. The region required for PHA-L binding lies within the boxed area. (**b**) The commonest form of O-linked glycosylation begins with the attachment of a single N-acetylgalactosamine residue to a serine (Ser) or threonine (Thr) residue of the polypeptide. This yields the Tn antigen. Tn can be extended in several ways. The addition of a beta1,3 linked galactose, catalysed by beta 1,3 galactosyltransferase (β1,3 GalT), yields the Thomsen-Friedenreich antigen. Alternatively, the addition of an alpha 2,6 linked sialic acid (NeuAc), catalysed by alpha 2,6 sialyltransferase (α2,6 ST), results in sialyl Tn. Key to symbols: shaded square = N-acetylglucosamine, GlcNAc; open circle = mannose, Man; shaded circle = galactose, Gal; open square = N-acetylgalactosamine, GalNAc; shaded diamond = sialic acid, NeuAc.

One of the many molecular changes that appears to be associated with successful metastasis is altered glycosylation of cancer cell glycoproteins. Perhaps this is unsurprising because more than half of all glycoproteins are glycosylated and their glycans have been demonstrated to be functional in a myriad of normal biological adhesion, communication, developmental, and identification mechanisms [76,77,78]. A detailed description of glycosylation lies beyond the scope of this review and the reader is directed to consult any one of many texts on the topic [79,80]. Some examples of the most intensively studied alterations in cancer cell glycosylation are given briefly below and are illustrated in Figure 2.

### 4.1. Increased Complexity and Branching of N-glycans

Glycosylation is mediated by glycosyltransferases. An increase in the activity of N-acetylglucosaminyltransferase V (GnT-V) expression in cancer cells causes an increase in the branching and elongation of N-glycans, particularly of β1, 6 branching [81], Figure 2a. This altered glycosylation can be detected by the binding of a lectin (a naturally occurring sugar-binding protein) derived from a bean, *Phaseolus vulgaris*, referred to as PHA-L. Lectins like PHA-L can be used in an analagous way to antibodies in immunohistochemistry to map the distribution and density of specific glycosylated structures on cells and tissues. Many studies have demonstrated an increase in β1, 6 branched N-glycans in cancers, including breast cancer, and that this is associated with the presence of metastases and consequent poor prognosis [82,83].

### 4.2. Truncation of O-glycans

O-linked glycans are usually shorter and more branched than N-linked glycans [84]. However, cancer cells typically synthesise even shorter and less branched O-glycans than normal cells [85]. 

The initial step in O-linked glycan synthesis is the addition of a single N-acetylgalactosamine (GalNAc) monosaccharide to the protein and is catalysed by a family of polypeptide-N-acetylgalactosamine transferases (GalNAc-Ts). The resulting structure, GalNAc-O-Ser/Thr, is referred to as Tn antigen (Figure 2b) and in normal cells, it is always elongated by the sequential addition of further monosaccharides. In cancer, strikingly, it is frequently left exposed. Its presence can be detected by the binding of another lectin, HPA, isolated from the Roman snail, *Helix pomatia*. There is substantial literature demonstrating that the presence of Tn antigen is associated with metastatic spread and consequent poor prognosis in breast and a range of other epithelial cancers [79]. This may be associated with cancer cell adhesion to the endothelial lining of vessels during the metastatic “cascade” described previously [86].

If Tn antigen is elaborated during O-linked glycosylation, one option is that a β1, 3 linked galactose can be added to yield the Thomsen-Friedenreich antigen (T or TF antigen, Galβ1-3GalNAcα-O-Ser/Thr) (Figure 2b). Whilst the Thomsen-Friedenreich antigen is, unlike Tn antigen, sometimes found on normal cells, it is unusual. It is frequently reported to be abundant on cancer cells and its presence has often been associated with metastasis and poor prognosis, possibly through mediating cancer cell adhesion to vascular endothelium [87]. 

### 4.3. Alterations in Sialylation

Alterations in sialylation, the addition of a sialic acid monosaccharide to terminate a glycan chain, is one of the most commonly reported alterations in glycosylation associated with cancer. There can be changes in the amount, type, and distribution of sialic acids on glycoproteins. Sialic acids can be involved in the immunogenicity, adhesion, and motility of cancer cells. They act to terminate the extension of glycan chains. For example, ST6 transferase-1(ST6GalNAc-1) acts to add a sialic acid to the Tn antigen GalNAc-O-Ser/Thr, described previously, to produce sTn antigen (Figure 2b). The presence of this structure has been associated with cancer cell adhesion and metastasis [88]. Conversely, silencing the glycosyltransferase ST6GalNAc-1 has been reported to inhibit proliferation and invasion of gastric carcinoma [89].

## 5. Changes in Cancer Cell Metastatic Capacity Post-Irradiation

Since metastasis is such a complex process involving a myriad of molecular mechanisms, it is unlikely that cancer cells that have been affected by irradiation—either directly or through bystander or abscopal effects—and survived would not exhibit some alterations in their metastatic ability. Indeed, there is considerable evidence for this in vitro and in pre-clinical studies [90] described below, and this may have implications for the development of radioresistance in tumours and consequent treatment failure. However, experimental results should be interpreted with caution as, clearly, in vitro and even in vivo models of cancer biology are not entirely reflective of the clinical situation and there is little convincing evidence that clinical use of radiotherapy to treat cancer results enhanced metastasis. Here, we briefly review some of the evidence in vitro, in vivo, and in clinical practice.

### 5.1. The Effect of Radiation on Angiogenesis

Radiation may have the effect of damaging/destroying both cancer cells and other cells in the microenvironment, including endothelial cells. The literature suggests that this may have complex and sometimes apparently contradictory effects, but ultimately may work to enhance invasiveness and metastasis of tumours. One simple example of this is that some cancer cells synthesise and secrete anti-angiogenic factors such as angiostatin and when the tumour is destroyed by radiation, a reduction in these factors induces angiogenesis and consequent growth of metastases. This was reported, for example, by Camphausen et al. in 2001 [91] in a mouse Lewis lung cancer model, after a fractionated regime of 10Gy per fraction for five fractions. Targeted radiation will also cause localised tissue damage and cell death and induce a hypoxic environment. Hypoxia stimulates angiogenesis and this will enhance both tumour growth, by re-establishing neovasculature, and metastasis, as briefly described previously. Enhanced establishment of lung metastases, for example, has been reported in a breast cancer mouse model following a single therapeutic dose of radiation to the thorax of the animals [92]. The reason for these effects seems to be that cancer cells damaged or killed by radiation secrete a range of soluble factors that stimulate angiogenesis and enhance cancer cell migration and invasion [93,94].

Radiation will also damage and destroy the endothelial cells that line vessels and, depending on the dose of radiation, this appears to have contradictory effects: generally, higher doses of 2–15Gy have been reported to have an anti-angiogenic effect, while lower doses of 0.5–0.8Gy appear to be pro-angiogenic [95,96]. However, the situation may be more complex than it appears. In a mouse model of breast cancer relapse after radiotherapy, a single high dose (20Gy) of radiation to the mammary gland reduced local vessel density, but when cancer cells were injected, there was an increase in invasion and tumours in lymph nodes, liver, and lungs [97].

### 5.2. The Effect of Radiation on Cancer Cell Motility, Invasion, and EMT

Paquette et al. (2007) showed that 20Gy irradiation of basement membrane components in an invasion assay led to increased invasion of MDA-MB-231 breast cancer cells through upregulation of several matrix metalloproteinases (MMP-2, MT1-MMP, and TIMP-2) [98]. An increase in invasive capacity following radiation, mediated through matrix metalloproteinase expression, has also been reported in a number of other cancer cells types, including melanoma [92], pancreatic cancer [99,100], glioma [101,102,103], rectal carcinoma [104], colon carcinoma, and osteosarcoma [105]. There is also evidence that irradiated cancer cells may secrete chemotactic factors that enhance the migration of neighbouring cells [106]. Radiation also promotes EMT in cancer cells, which would be consistent with this enhanced invasive phenotype. For example, Kawamoto et al. (2012) demonstrated enhanced migration and invasion in colorectal cancer cells after low dose (single 2.3Gy dose) irradiation, and this was clearly associated with both molecular and phenotypic changes characteristic of EMT. Furthermore, they observed molecular changes consistent with EMT in tumour samples taken from colorectal cancer patients undergoing radiotherapy [107].

Owing to the complexity of metastatic mechanisms briefly reviewed earlier, the underlying molecular and metabolic changes induced by exposure to radiation during this process are also extremely complex and go beyond the scope of this review. The topic is explored in depth by Lee et al. (2017) who examine the underlying molecular mechanisms of radiation-induced EMT, more general alterations in the tumour microenvironment and oncogenic metabolism, and the potential role of cancer stem cells [108].

### 5.3. The Effect of Radiation on Normal and Cancer Cell Glycosylation

Alterations in cellular glycosylation are common in cancers and may have functional significance, as described previously. However, very little is known about the effect of radiation on cellular glycosylation processes in general or cancer cell glycosylation in particular.

Iizuka et al. (2018) recently looked at global changes in glycosylation of serum glycoproteins from mice after whole body radiation. Their most significant finding was an increase in alpha 2,3 linked sialic acid and a decrease in alpha 2,6 linked sialic acid, and also changes in glycans bearing N-acetylglucosamine (GlcNAc). They interpreted these changes as being related to an acute phase response and inflammation following radiation damage [109]. On the other hand, Chaze et al. (2013), in a similar study, observed a decrease in bi-antennary and increase of tri-, tetra-, and penta-antennary structures on N-glycans, as well as an increase in fucosylation and sialylation [81]. Changes in glycosylation of serum glycoproteins have also been reported in patients undergoing radiotherapy. For example, Toth et al. (2016) analysed glycosylation of seven common serum glycoproteins in head and neck cancer patients undergoing radiotherapy, using mass spectrometry, over a period of up to 15 months. They found varied and complex changes in the glycoforms of the proteins over time, which appeared to result from the effects of radiation and which returned to “normal” over time [110]. Such changes undoubtedly represent cellular responses and adaptations following injury, damage, and cell death due to radiation, but the functional implications of such changes to cancer biology and metastasis, if any, are not understood.

We described previously how cancer cells may utilise similar mechanisms to leukocytes in order to adhere to the endothelium and extravasate during metastasis. This interaction between leukocytes and endothelium is part of the normal inflammatory response, which is also implicated in cancer biology (and a description of this goes beyond the scope of this review), and may be affected by radiation. Jaillett et al. (2017) specifically analysed glycosylation changes in endothelial cells following radiation exposure and their functional consequences. They found an increase in high-mannose or oligomannose type N-glycans (these are glycans where the trimannosyl core is extended by mannose residues exclusively) and an increase in monocyte adhesion to endothelial cells, but were unable to demonstrate that the glycans were functional in this adhesion. They suggested that alterations in glycosylation may be a mechanism by which organisms regulate cell adhesion during inflammation, recovery, and healing after radiation exposure [111].

Very little is known about potential changes in cancer cell glycosylation related to cell behaviour following radiation. Intriguingly, however, Jaillett et al. (2017) showed overexpression of several polypeptide-N-acetylgalactosamine (GalNAc) transferases (GalNAc-Ts), the family of genes that mediate the attachment of the first GalNAc in O-linked glycosylation, by endothelial cells, in mouse intestine after radiation exposure [111]. As described previously, cancer cells frequently exhibit an increase in truncated O-glycans consistent with this type of general up-regulation in O-linked glycosylation. The presence of these GalNAc-glycans, especially of unelaborated O-linked GalNAc (the Tn antigen), are associated with enhanced metastasis and poor prognosis and have been functionally implicated in cancer cell adhesion to endothelium [79,86], described previously. Thus, these findings may indicate a mechanism by which cancer cell metastasis might be enhanced following radiation.

There is also some evidence that increased beta 1,6 branching of N-glycans, a common feature of cancer cells and known to be associated with metastasis and poor prognosis in breast and other cancers [82,83], as described previously, may be linked to radioresistance, at least in some cancer types. When radioresistance is induced in human nasopharangeal cancer cells by exposing them repeatedly to low levels of radiation, they express higher levels of N-acetylglucosaminyltransferase V (GnT-V) and of beta 1,6 branched glycans. Conversely, knocking down GnT-V in these cells reduces beta 1,6 branched glycans and restores radiosensitivity [112]. Consistent observations have been reported in glioma where clinical samples were shown to contain high levels of beta 1,6 branching glycans in comparison to benign astrocytomas or non-neoplastic brain, and this correlated with radioresistance. Moreover, knockdown of GnT-V and consequent reduction in beta 1,6 branched glycans was associated with greater sensitivity to radiation in glioma cell culture [113]. In both of the studies cited here, inhibitors of N-glycan synthesis (swainsonine and tunicamycin, respectively) also enhanced radiosensitivity in the cancer cells.

### 5.4. Metastasis Following Radiotherapy in the Clinical Setting

Standard treatment for primary breast cancer typically includes surgery to remove the primary tumour with radiotherapy to residual breast tissue or chest wall and axilla to control locoregional recurrence. The major cause of treatment failure and death in patients, however, is distant metastatic disease, reviewed earlier. This may only manifest itself years and sometimes decades later once micrometastases have adapted to their new environment, flourished, and become clinically detectable. Treatment regimes will have evolved significantly in the meantime, thus calling into question the relevance of any findings to modern practice. Moreover, at the present time, it is impossible to distinguish between metastases that have resulted from micrometastases that were present at the time of initial diagnosis and treatment and those that potentially may have been induced or enhanced by treatments, including radiotherapy. This situation may change as technologies to study the genetics and biology of individual tumours increases. 

In the meantime, it is possible to look for evidence as to whether patients treated for primary breast and other cancers who have received radiotherapy subsequently suffer increased distant metastasis, and such evidence is generally lacking. This situation is well represented by a very comprehensive systematic review of radiotherapy effects in breast cancer treatment reported by Rutqvist et al. (2003). It included data from more than 40,000 patients and looked at radiotherapy after mastectomy as part of breast-conserving surgery and in patients given supplementary radiation (radiation boost or radiotherapy to the internal mammary chain). While for the reasons stated previously, these authors, as others, did not seek evidence for radiotherapy inducing metastasis specifically, they did report on disease free and overall survival of patients receiving and not receiving such treatment, so comparisons can be made. The report contains no suggestion that patients receiving radiotherapy as part of their primary treatment experienced enhanced distant metastasis compared to those that did not. For example, radiotherapy actually improved overall survival in at least some mastectomy patients and resulted in those treated by conservative surgery experiencing comparable survival to mastectomy patients [114]. There is also strong evidence from meta-analysis of randomised controlled trials that radiotherapy to axillary lymph nodes in stage I to III breast cancer patients reduces the risk of distant metastasis rather than enhances it and thus improves survival at 10 years [115]. 

In spite of the clear benefit in primary breast cancer therapy, there is some clinical evidence of a potentially metastasis-promoting effect of radiotherapy in the clinical situation, in that non-small cell lung cancer (NSCLC) patients receiving radiotherapy exhibit increased numbers of circulating tumour cells (CTCs) that can be shown to be derived from the irradiated tumour [116]. Other studies have shown similar results of increased numbers of CTCs in the blood of patients receiving radiotherapy for primary NSCLC [117] and 3 months after radiotherapy for prostate cancer [118], albeit in very small patient samples. The significance of these observations is yet unknown and a greater understanding of their relevance and that of other potential mediators of radiation-induced cell signalling, such as through exosomes, considered in the next section, may ultimately result in the potential to further improve radiotherapy efficacy.

## 6. Exosomes and the Effect of Post-Irradiation Exosomes on Cancer Cells 

### 6.1. Exosomes as Mediators of Cell–Cell Communication 

**Figure 3:**
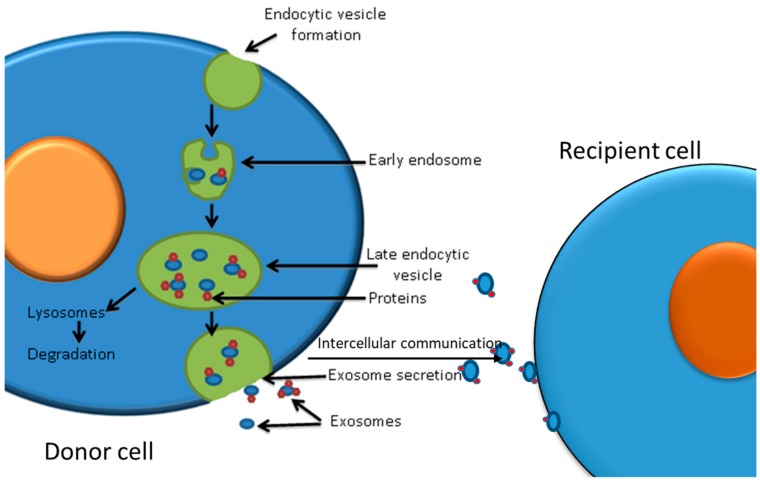
Exosome biogenesis, secretion, and cell communication via exosomes.

Exosomes are 30–150 nm small nanovesicles, which are endosomal in origin, see Figure 3 [119]. They contain proteins, microRNAs, mRNAs, lipids, mitochondrial DNA, and genomic DNA [120] and are secreted by normal and pathological cells into the microenvironment. They are therefore detected in a variety of biofluids such as plasma, urine, saliva, ascites fluid, breast milk, and semen [121]. Likewise, cancer cells release exosomes into the tumour microenvironment and peripheral blood [122]. Exosomes are mediators of cell–cell communication as they can be received and taken up by a wide range of cell types (Figure 3), altering cells’ molecular profile, signalling pathways, and gene regulation [123]. They can also transfer genetic information to the recipient cells, promoting epigenetic changes [124].

### 6.2. Exosomes Involvement in Enhancing Tumour Progression and Metastasis

Tumour cells communicate with each other and with neighbouring stromal cells through exosomes and share biological information. Therefore, exosomes can enhance tumourigenesis, angiogenesis, immunosuppression, and the development of metastasis, causing resistance to therapies [125,126]. There is also evidence that exosomes derived from irradiated cancer cells have the ability to induce genomic instability in recipient non-irradiated cells, leading to relapse [127].

Exosomes derived from cancer cells contain proteins, nucleic acids, lipids, and other substances derived from their parental cells [128]. Exosomes have been well established to be involved in all cancer stages [129,130,131]. Exosome-mediated signal transduction and intercellular communication can play a crucial role in the development, progression, and treatment of cancer [132]. Zhang et al. have studied the effects of exosomes derived from gastric cancer cells in the liver microenvironment; they found that epidermal growth factor receptor carried and transferred by exosomes has the ability to enhance liver-specific metastasis [133]. Moreover, miRNA molecules of exosomes derived from breast cancer patients can promote transformation and tumour formation in recipient normal epithelial cells [134].

Another study by Dioufa et al. (2017) showed that priming the hepatic niche with exosomes exuded from MDA-231 breast cancer cells facilitated seeding of the cancer cells in the liver. Intriguingly, the same study also showed a difference in a certain set of miRNA contents in the tumour-derived exosomes compared with exosomes from normal cells (which happened to be a set of miRNAs involved in epithelial cell differentiation) [135]. It was also shown that bone-derived exosomes can stimulate proliferation in tumour cells, as well as mediate communication between cancer cells and bone cells [136]. These strands of evidence highlight how exosomes can aid in metastasis by helping tumour cells to adapt to a different microenvironment. 

### 6.3. The Potential of Exosomes to Enhance the Effectiveness of Radiation Therapy

Much evidence has shown that exosomes are associated with cancer pathology and neurodegenerative diseases [137]; however, less is known about their potential involvement in radiation biology. Although there are only few publications covering radiation and exosomes, these have been promising in opening avenues for a new and better understanding of cancer and other diseases and raise questions as to whether it might be possible to harness the effects of exosomes to enhance radiotherapy success.

It has been proposed that exosome release is first activated by stress-inducible pathways in which increased expression of transmembrane protein tumour suppressor-activated pathway 6 (TSAP6) is stimulated by the damage-induced p53 transcription factor [138]. Once released from the donor cell, exosomes are transferred to and enter recipient cells through such processes as phagocytosis and membrane fusion. Once the radiation-induced exosomes have entered the target cell, specific processes in the target cell are activated [139]. It is well established that irradiated cells can produce factors, such as exosomes, that influence unirradiated neighbouring cells, a phenomenon recognised as the radiation-induced bystander effect (RIBE) [140]. In RIBE, non-irradiated cells exhibit radiation-type effects as a result of having some form of communication with those cells that have had exposure to radiation. Recent studies showed that exosomes derived from irradiated cells disseminate ionising radiation-induced effects to the unirradiated cell and may cause functional responses in recipient cells [141].

Ionising radiation has been shown to increase the release of exosome release by a variety of cell lines [127,140,142]. Research has also shown that exosome release is influenced by timing and dose of irradiation. For example, a study using a glioblastoma cell line (U87MG) irradiated with 2, 4, 6, and 8 Gy of X-rays showed an increase in exosome secretion in a dose-dependent manner 24 h post-irradiation (PI) [127]. In the latter study, irradiated cells-derived exosomes were labelled with PKH26 red fluorescing dye and incubated with fresh recipient cells. Data showed that exosomes were found in the cytoplasm and on the surface of recipient cells, suggesting that exosomes secreted from cancer cells can be received by neighbouring cells following irradiation.

A study by Al-Mayah et al. (2015) has also shown that exosomes from irradiated cells are able to deliver RNA and induce RIBE, as well as the delayed effects of genomic instability (GI). In this study, media from 2 Gy x-ray irradiated breast cancer cells (MCF7) as well as their progeny were collected. Then exosomes from these media were extracted, purified, and transferred to healthy fresh non-irradiated cells. DNA damage was apparent in early and delayed recipient cell populations, showing that exosomes released by directly-irradiated cells and their progeny play an important role in mediating the non-targeted effects of IR [142]. In another study in which human keratinocyte cells (HaCaT) were irradiated with 0.005, 0.05, and 0.5 Gy γ-rays, exosomes were then extracted and removed from irradiated cell conditioned media (ICCM). Then, ICCM without exosomes (exosomes-depleted media) was transferred to fresh non-irradiated cells; however, a reduction in cell death, calcium influx, and reactive oxygen species were observed in the recipient cells compared to the completed ICCM media (ICCM with exosomes), suggesting that exosomes are significantly involved in the ionising radiation-induced bystander effects [124]. 

Further, cancer-derived exosomes can trigger an immune response, which studies have revealed can be either suppressive or enhance immune activities. For example, Wen et al. (2016) determined that exosomes derived from breast cancer cells suppress an immune response by directly inhibiting T-cell proliferation and decreasing natural killer cell cytotoxicity, therefore promoting metastasis [143]. Contrastingly, studies involving exposing tumour cells to stress have shown improvements in anti-tumour immunity. For example, Dai et al. (2005) showed that exosomes released from heat-stressed carcinoembryonic antigen (CEA)-positive tumour cells have high immunogenicity [144]. Another study showed that following exposure to X-irradiation, exosomes originating from tumour cells contain dsDNA that activated stimulation of interferon signalling in recipient cells, thereby causing a protective anti-tumour T-cell response [141]. 

A number of studies have demonstrated that exosomes derived from radiation-exposed cells have biological effects on recipient cancer cells [140,145,146,147,148,149]. A study by Golden et al. showed that metastatic spread of melanoma cells is frequently mediated by mesenchymal stem cell exosomes following irradiation, in which exosomes can be considered as mediators of potential cross-talk between cancer cells and surrounding stroma [146]. Freudenmann et al. have also documented that L-Plastin molecules delivered by exosomes can lead to clonogenic and mitogenic activity in normal and cancer cells within the microenvironment of the tumour. They have found that L-Plastin can be inhibited by IR, which may in part explain the impact of RT in cancer control [147].

## 7. Conclusions

The breast is particularly susceptible to radiation at certain points during development and radiation exposure can increase breast cancer risk. Radiation can also be used to treat both primary and metastatic breast cancer and is proven to be highly effective in controlling locoregional recurrence when used alongside surgery to treat primary breast cancer. Radiotherapy operates through several mechanisms, including through stimulation of the immune system. However, the situation may be far more complex than has been previously realised. In this paper, we review the accumulating evidence from in vitro and in vivo studies for increased metastatic potential in cancer cells that either survive IR or are exposed to exosomes released from irradiated cells. Whilst there is little evidence that radiotherapy to treat primary breast cancer results in enhanced metastasis in the clinical setting and there is ample evidence of its therapeutic benefits, a better understanding of its biological effects may have implications for cancer diagnosis and therapy using IR. By better understanding the molecular mechanisms involved in post-irradiation effects, including exosome-mediated cell–cell communication post-irradiation, there is the potential of actually enhancing the therapeutic effects of radiation therapy.
